# On the Performance Evaluations of Cooperative Retransmission Scheme for Cell-Edge Users of URLLC in Multi-Carrier Downlink NOMA Systems

**DOI:** 10.3390/s21217052

**Published:** 2021-10-24

**Authors:** Won-Jae Ryu, Jae-Woo Kim, Soo-Young Shin, Dong-Seong Kim

**Affiliations:** 1 ICT Convergence Research Center, Kumoh National Institute of Technology, Gumi 39177, Gyeongbuk, Korea; wj0828@kumoh.ac.kr (W.-J.R.); jaewookim@kumoh.ac.kr (J.-W.K.); 2 Department of IT Convergence Engineering, Kumoh National Institute of Technology, Gumi 39177, Gyeongbuk, Korea

**Keywords:** cell-edge user (CEU), non-orthogonal multiple access (NOMA), retransmission, ultra-reliable and low-latency communication (URLLC), cooperative transmission

## Abstract

Non-orthogonal multiple access (NOMA) has a key feature that the cell-center user (CCU) has prior information about the messages of the cell-edge user (CEU) in the same user-pair. It means that CCU can be used for retransmission when the CEU requests retransmission. As ultra-reliability and low-latency communication (URLLC) requires high-reliability constraints (e.g., 99.999%), using CCU for retransmission can be useful to satisfy the reliability constraint. In this study, to ensure the reliability of CEU, cooperative retransmission (CR) scheme for downlink NOMA systems is proposed. And the CR scheme is evaluated with Block error rate (BLER) considering reliability and with packet loss rate (PLR) in terms of reliability and latency constraints. And the evaluation results showed that the proposed CR scheme can satisfy the target BLER for URLLC low SNR compared to the conventional retransmission scheme, and showed the improved PLR compared to the conventional retransmission scheme in low SNRs.

## 1. Introduction

Ultra-reliability and low-latency communication (URLLC) is one of the requirements of the fifth-generation (5G) new radio. The URLLC service was introduced in applications requiring stringent latency constraints and high-reliability constraints [1].

Non-orthogonal multiple access (NOMA) can be considered a solution for URLLC due to its high spectral efficiency and ability to reduce latency by superposing signals of users in a user-pair at the same time and same frequency [2]. Therefore, several types of studies have been conducted to investigate URLLC via NOMA.

Kotaba et al. introduced the concept of NOMA hybrid automatic repeat request that superposes transmission and retransmission to reduce using resource blocks in uplink streams [3]. Ryu and Shin studied the power allocation in the downlink NOMA system based on the finite blocklength regime [4]. Doğan et al. proposed a novel non-orthogonal resource-sharing scheme based on orthogonal frequency-division multiple access index modulations to achieve tight latency in URLLC [5]. Ren et al. jointly optimized resource blocks and power allocation to minimize the decoding error probability to achieve URLLC requirements with cooperative-NOMA in a factory automation scenario [6]. Amjad and Musavian analyzed the performance limitations of NOMA for URLLC in terms of queuing delay, error rate, and packet size [7]. Rai et al. proposed a NOMA-enabled fog-cloud structure for enhanced mobile broadband (eMBB) and URLLC traffic in accordance with different aspects of high- and low-density networks [8]. Imtiaz Jaya and Hossain proposed two user clustering techniques in uplink NOMA systems to meet latency constraints of time stringent services via resource slicing [9]. Do et al. proposed a cooperative relay scheme for cell-edge users (CEUs) in NOMA systems [10]. Most of the above studies focused on the optimization of resources in NOMA systems. Even though the performance improvement of the CEUs was addressed in [10], it was studied in terms of capacity improvement. NOMA has a key feature that the cell-center user (CCU) has prior information about the messages of the CEU. Therefore, if the CEU asks for retransmission, Base station (BS) and CCU can retransmit the signal and the CEU can do maximum ratio combing (MRC). Liu et al. investigated a cooperative NOMA to compare various relaying schemes in terms of outage proability and average throughputs [11]. Kara and Kaya analyzed error performance of cooperative NOMA [12] and proposed threshold-based selective cooperative NOMA [13]. Wei and Wong proposed a series of novel coordination scheme for multi-cell downlink communication [14]. However, above studies only consider performance evaluation of cooperative NOMA, not directly related to URLLC. In this study, the cooperative relay system in NOMA is adopted as a retransmission scheme to improve performance in terms of the reliability and latency constraints of URLLC for CEUs. The contributions of this study are as follows:

- Suggesting the cooperative relay as a cooperative retransmission (CR) scheme in downlink NOMA systems to improve the reliability of CEUs for URLLC.
- Providing an analytical model and performance evaluation for block error rate(BLER) in terms of URLLC reliability.
- Providing performance evaluations of packet loss rate(PLR) for the CR scheme considering the latency constraint of URLLC system with limited resource blocks.
- Showing that the CR scheme outperforms the conventional retransmission scheme in terms of URLLC constraints.

This study is organized as follows: The system model is described in Section 2. CR scheme and analytical models are described in Section 3. In Section 4, the simulation description and results are presented. Finally, in Section 5, the conclusions of the study and the scope of future work are discussed.

## 2. System Model

**Figure 1:**
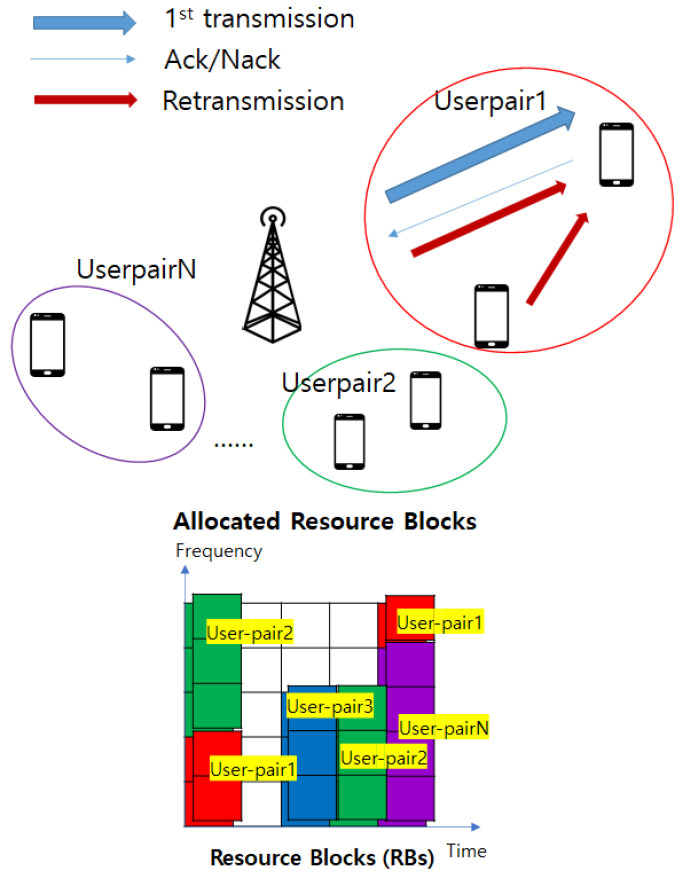
System model.

**Figure 2:**
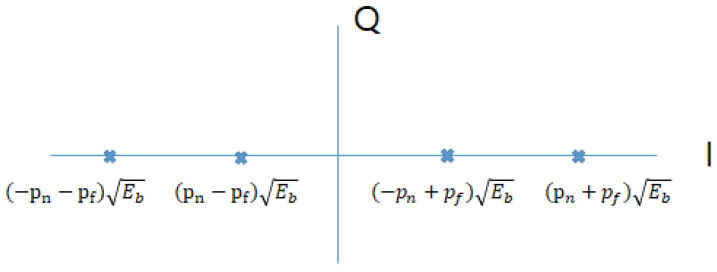
Constellation of BPSK NOMA.

In the system model illustrated in Figure 1, the downlink multicarrier NOMA system is adopted. It comprises one BS, and multiple user-pairs consisting of two users, the CCU and the CEU, where each device is equipped with a single antenna. As the CCU should do the successive interference cancellation (SIC), the CCU can know the signal for the CEU. This implies that, when retransmission is requested from the CEU, the CCU can also respond to the retransmission with the BS. For simplicity, binary phase-shift keying (BPSK) is adopted for both the CCU and the CEU. As this study focuses on the improvement of reliability for CEU, the block error rate (BLER) must be derived for the CEU. In this section, the system model is described based on BLER. Before deriving the BLER for the CEU, the bit error rate (BER) must be determined. Figure 2 shows the constellation of BPSK-based NOMA. To derive p(e), the BER of CEU:

(1) $$\begin{array}{c} p\left(e\right)=p\left(s_{0,0}\right)p\left(e|s_{0,0}\right)+p\left(s_{0,1}\right)p\left(e|s_{0,1}\right)\\ +p\left(s_{1,0}\right)p\left(e|s_{1,0}\right)+p\left(s_{1,1}\right)p\left(e|s_{1,1}\right), \end{array}$$

(2) $$p\left(s_{0,0}\right)=p\left(s_{0,1}\right)=p\left(s_{1,0}\right)=p\left(s_{1,1}\right)=\frac{1}{4},$$

(3) $$p\left(e|s_{1}\right)=p\left(e|s_{0}\right).$$

$p(s_{x,y})$ denotes the probability when each bit for the CEU and the CCU is *x* and *y*, respectively. Subsequently, $p(e|s_{0})$, the BER with the channel coefficient *h* in the fading channel, when the bit for the CEU is 0, is as follows:

(4) $$p\left(e|s_{0}\right)=p\left(e|s_{0,0}\right)+p\left(e|s_{0,1}\right).$$

$s_{0,0}$ and $s_{0,1}$ are $\left(-p_{n}-p_{f}\right)\sqrt{E_{b}}$ and $\left(p_{n}-p_{f}\right)\sqrt{E_{b}}$ respectively as shown in Figure 2. $p_{n}$ is the power allocation ratio for the CCU, $p_{f}$ is the power allocation ratio for the CEU. $\left(-p_{n}-p_{f}\right)\sqrt{E_{b}}$ means the bit for near user and the bit for far user are 0, and 0, respectively, $\left(p_{n}-p_{f}\right)\sqrt{E_{b}}$ means the the bits are 1 and 0, $\left(-p_{n}+p_{f}\right)\sqrt{E_{b}}$ means the bits are 0 and 1, and finally $\left(p_{n}+p_{f}\right)\sqrt{E_{b}}$ means the bits are 1 and 1.

(5) $$\begin{array}{c} p\left(e|s_{0}\right)=\frac{1}{\sqrt{\pi N_{0}}}\int_{-\infty }^{0}e^{\frac{-{\left(y-\left(-p_{n}-p_{f}\right)h\sqrt{E_{b}}\right)}^{2}}{N_{0}}}dy\\ +\frac{1}{\sqrt{\pi N_{0}}}\int_{-\infty }^{0}e^{\frac{-{\left(y-\left(p_{n}-p_{f}\right)h\sqrt{E_{b}}\right)}^{2}}{N_{0}}}dy. \end{array}$$

*h* is the channel coefficient, $E_{b}$ is energy per bit, and $N_{0}$ is noise power spectral density.

(6) $$\begin{array}{c} p\left(e|s_{0}\right)=\frac{1}{\sqrt{\pi }}\int_{\left(p_{n}+p_{f}\right)h\sqrt{\frac{E_{b}}{N_{0}}}}^{\infty }e^{-z^{2}}dz\\ +\frac{1}{\sqrt{\pi }}\int_{\left(p_{f}-p_{n}\right)h\sqrt{\frac{E_{b}}{N_{0}}}}^{\infty }e^{-z^{2}}dz, \end{array}$$

(7) $$\begin{array}{c} p\left(e|s_{0}\right)=\frac{1}{2}erfc\left(\left(p_{n}+p_{f}\right)h\sqrt{\frac{E_{b}}{N_{0}}}\right)\\ +\frac{1}{2}erfc\left(\left(p_{f}-p_{n}\right)h\sqrt{\frac{E_{b}}{N_{0}}}\right). \end{array}$$

Herein, $\frac{E_{b}}{N_{0}}$, signal to noise ratio (SNR), is expressed as μ. Finally, as shown in (1)–(4), the BER for the CEU p(e) will be:

(8) $$p\left(e\right)=\frac{1}{4}\left(erfc\left(\left(p_{n}+p_{f}\right)h\sqrt{\mu }\right)+erfc\left(\left(p_{f}-p_{n}\right)h\sqrt{\mu }\right)\right),$$

$\epsilon_{1}$, the BLER of the first transmission based on (8), is

(9) $$\epsilon_{1}=1-{\left(1-p\left(e\right)\right)}^{n},$$

where *n* denotes the number of bits in the resource block. According to Figure 1, the retransmission occurs after the first transmission. At this time, there is no power level differentiation for superposing data transmission. Therefore, the BLER model for retransmission $\epsilon_{2}$ is based on the BPSK model as follows:

(10) $$p_{bpsk}\left(e\right)=\frac{1}{2}erfc\left(h\sqrt{\mu }\right),$$

(11) $$\epsilon_{2}=1-{\left(1-p_{bpsk}\left(e\right)\right)}^{n},$$

where, $p_{bpsk}\left(e\right)$ is the BER of BPSK signal.

Finally, the final BLER α, which considers both the first transmission $\epsilon_{1}$ and retransmission $\epsilon_{2}$, is

(12) $$\alpha =\epsilon_{1}\times \epsilon_{2}.$$

Equation (12) means that the superposed signal for both CCU and CEU is transmitted when the first transmission comes out, and the signal for only the CEU is transmitted when the retransmission is requested from the CEU.

## 3. Cooperative Retransmission Scheme

In this section, the CR scheme and analytical model are described. The first subsection describes the BLER of CR scheme in the ideal scenario, which assumes perfect channel state information (P-CSI) and perfect-SIC (P-SIC). The second subsection describes the proposed scheme in the practical scenario, which assumes imperfect-CSI (I-CSI) and imperfect-SIC (I-SIC).

### 3.1. BLER of Cooperative Retransmission Scheme

#### 3.1.1. Ideal Scenario

P-CSI and P-SIC are adopted for the ideal scenario. In the conventional retransmission scheme, only the BS sends retransmission signals. Instead, in the proposed NOMA system, the CCU can be used for retransmission to the CEU. The CCU perform SIC to obtain their own data. Therefore, the CCU should know the signal for the CEU, indicating that the CCU can also transfer retransmission to the CEU. Both the CCU and the BS can perform retransmission when CEUs request retransmission, as shown in Figure 1. For the first transmission, the BLER is expressed as follows:

(13) $$\begin{array}{c} \epsilon_{1}=1-{\left(1-\int_{0}^{\infty }\underset{\mathrm{Rayleigh}\;\mathrm{fading}\;\mathrm{PDF}}{\underset{︸}{\frac{h}{\sigma^{2}}e^{\frac{-h^{2}}{2\sigma }}}}\underset{\mathrm{Eq}\;(8)}{\underset{︸}{p(e)}}dh\right)}^{n}, \end{array}$$

where σ is the standard deviation of fading channel.

To express (13) in closed-form [15], we take the integral part as follows:

(14) $$P\left(e\right)=\int_{0}^{\infty }\frac{h}{\sigma^{2}}e^{\frac{-h^{2}}{2\sigma }}p\left(e\right)dh,$$

(15) $$\begin{array}{c} P\left(e\right)=\frac{1}{4}\int_{0}^{\infty }\frac{h}{\sigma^{2}}e^{-\frac{h^{2}}{2\sigma^{2}}}(erfc\left(\left(p_{n}+p_{f}\right)h\sqrt{\mu }\right)\\ -(erfc\left(\left(p_{f}-p_{n}\right)h\sqrt{\mu }\right))dh, \end{array}$$

(16) $$\begin{array}{c} P\left(e\right)=\frac{1}{4}(2-\sqrt{\frac{2\sigma^{2}{\left(p_{n}+p_{f}\right)}^{2}\mu }{1+2\sigma^{2}{\left(p_{n}+p_{f}\right)}^{2}\mu }}\\ -\sqrt{\frac{2\sigma^{2}{\left(p_{f}-p_{n}\right)}^{2}\mu }{1+2\sigma^{2}{\left(p_{f}-p_{n}\right)}^{2}\mu }}). \end{array}$$

Therefore:

(17) $$\epsilon_{1}=1-{\left(1-P\left(e\right)\right)}^{n}.$$

Subsequently, to obtain the BLER for retransmission, a bivariate rayleigh fading channel is considered in the case of simultaneous retransmission from the BS and CCU to the CEU. To derive the probability density function (PDF) of the bivariate rayleigh fading channel is as follows:

(18) $$p_{df}\left(x,y,\sigma_{x},\sigma_{y}\right)=\frac{xy}{\sigma_{x}^{2}\sigma_{y}^{2}}e^{\frac{-x^{2}}{2\sigma_{x}^{2}}+\frac{-y^{2}}{2\sigma_{y}^{2}}},$$

where *x* is the channel state between the BS and the CEU, *y* is the channel state between the CCU and CEU, $\sigma_{x}$ is the standard deviation for *x*, and $\sigma_{y}$ is the standard deviation for *y*. We assume that the BS and CCU retransmit signals at the same power level per one subcarrier. It means that the total power consumption of CCU is not much as BS, as the CCU only uses parts of whole subcarriers for retransmission. And retransmission is not always happened. Therefore, we can assume that the CCU can be used for retransmission.

For the side of the CEU, retransmission from the BS and CCU are considered as the combined channel coefficient, as the CEU can do MRC. The channel coefficient *x* between the BS and the CEU, and the channel coefficient *y* between the CCU and the CEU are combined as *r* when the retransmission is requested. Therefore, PDF on *r* should be derived as follows:

(19) $$r\geq 0,\;x\geq 0,\;y\geq 0,\;r^{2}=x^{2}+y^{2},$$

(20) $$y=\sqrt{r^{2}-x^{2}},\;dy=\frac{x}{\sqrt{r^{2}-x^{2}}}dx,$$

(21) $$P_{df}\left(x,\sigma_{x},\sigma_{y}\right)=\frac{x^{2}}{\sigma_{x}^{2}\sigma_{y}^{2}}e^{\frac{\left(\sigma_{x}^{2}-\sigma_{y}^{2}\right)x^{2}-\sigma_{x}^{2}r^{2}}{2\sigma_{x}^{2}\sigma_{y}^{2}}}dxdx,$$

(22) $$\frac{dr}{dx}=\frac{-x}{\sqrt{x^{2}+y^{2}}},\;dx=\frac{-\sqrt{x^{2}+y^{2}}}{x}dr=\frac{-r}{x}dr,$$

(23) $$P_{df}\left(x,r,\sigma_{x},\sigma_{y}\right)=\frac{x^{2}}{\sigma_{x}^{2}\sigma_{y}^{2}}e^{\frac{\left(\sigma_{x}^{2}-\sigma_{y}^{2}\right)x^{2}-\sigma_{x}^{2}r^{2}}{2\sigma_{x}^{2}\sigma_{y}^{2}}}\frac{-r}{x}drdx,$$

(24) $$P_{df}\left(x,r,\sigma_{x},\sigma_{y}\right)=\frac{-rx}{\sigma_{x}^{2}\sigma_{y}^{2}}e^{\frac{\left(\sigma_{x}^{2}-\sigma_{y}^{2}\right)x^{2}-\sigma_{x}^{2}r^{2}}{2\sigma_{x}^{2}\sigma_{y}^{2}}}drdx,$$

(25) $$x=rsin\theta ,\;dx=-rcos\theta d\theta ,\;0\leq \theta \leq \frac{\pi }{2},$$

(26) $$P_{df}\left(x,r,\sigma_{x},\sigma_{y}\right)=\int \int_{0}^{\frac{\pi }{2}}\frac{r^{3}sin2\theta }{2\sigma_{x}^{2}\sigma_{y}^{2}}e^{\frac{\left(\sigma_{x}^{2}-\sigma_{y}^{2}\right)x^{2}-\sigma_{x}^{2}r^{2}}{2\sigma_{x}^{2}\sigma_{y}^{2}}}d\theta dr,$$

(27) $$p_{df}\left(r\right)=\int_{0}^{\frac{\pi }{2}}\frac{r^{3}sin2\theta }{2\sigma_{x}^{2}\sigma_{y}^{2}}e^{\frac{\left(\sigma_{x}^{2}-\sigma_{y}^{2}\right)r^{2}sin^{2}\theta -\sigma_{x}^{2}r^{2}}{2\sigma_{x}^{2}\sigma_{y}^{2}}}d\theta ,$$

(28) $$p_{df}\left(r\right)=\frac{r^{3}}{2\sigma_{x}^{2}\sigma_{y}^{2}}\int_{0}^{\frac{\pi }{2}}sin2\theta e^{\frac{\left(\sigma_{x}^{2}-\sigma_{y}^{2}\right)r^{2}sin^{2}\theta -\sigma_{x}^{2}r^{2}}{2\sigma_{x}^{2}\sigma_{y}^{2}}}d\theta ,$$

(29) $$sin^{2}\theta =t,\;d\theta =\frac{dt}{sin2\theta },$$

(30) $$p_{df}\left(r\right)=\frac{r^{3}}{2\sigma_{x}^{2}\sigma_{y}^{2}}\int_{0}^{1}e^{\frac{\left(\sigma_{x}^{2}-\sigma_{y}^{2}\right)r^{2}t-\sigma_{x}^{2}r^{2}}{2\sigma_{x}^{2}\sigma_{y}^{2}}}dt.$$

Therefore, the bivariate rayleigh fading channel PDF for *r* is:

(31) $$p_{df}\left(r\right)=\frac{r}{\sigma_{x}^{2}-\sigma_{y}^{2}}\left(e^{\frac{-r^{2}}{2\sigma_{x}^{2}}}-e^{\frac{-r^{2}}{2\sigma_{y}^{2}}}\right).$$

The PDF of channel gain from both the BS and the CCU to the CEU can be modeled as $p_{df}\left(r\right)$ in (31). To obtain $\epsilon_{2}$, the BLER for retransmission is as follows:

(32) $$\begin{array}{c} \epsilon_{2}=1-{\left(1-\int_{0}^{\infty }\underset{\mathrm{Eq}\;(31)}{\underset{︸}{p_{df}\left(r\right)}}\underset{\mathrm{Eq}\;(10)}{\underset{︸}{p_{bpsk}\left(e\right)}}dr\right)}^{n}. \end{array}$$

To derive (32) into the closed-form:

(33) $$\begin{array}{c} \int_{0}^{\infty }p_{df}\left(r\right)p_{bpsk}\left(e\right)dr\\ =\int_{0}^{\infty }\frac{r}{\sigma_{x}^{2}-\sigma_{y}^{2}}\left(e^{\frac{-r^{2}}{2\sigma_{x}^{2}}}-e^{\frac{-r^{2}}{2\sigma_{y}^{2}}}\right)\frac{1}{\sqrt{\pi }}\int_{r\sqrt{\mu }}^{\infty }e^{-t^{2}}dtdr. \end{array}$$

We take the part of $e^{\frac{-r^{2}}{2\sigma_{x}^{2}}}$ in (33) as follows:

(34) $$\begin{array}{c} \frac{1}{\left(\sigma_{x}^{2}-\sigma_{y}^{2}\right)\sqrt{\pi }}\int_{0}^{\infty }re^{\frac{-r^{2}}{2\sigma_{x}^{2}}}\int_{r\sqrt{\mu }}^{\infty }e^{-t^{2}}dtdr. \end{array}$$

Substitution proceeds as follows:

(35) $$u=\frac{t}{r\sqrt{\mu }},\;\frac{du}{dt}=\frac{1}{r\sqrt{\mu }},$$

(36) $$\frac{\sqrt{\mu }}{\left(\sigma_{x}^{2}-\sigma_{y}^{2}\right)\sqrt{\pi }}\int_{1}^{\infty }\int_{0}^{\infty }r^{2}e^{-\left(\frac{1}{2\sigma_{x}^{2}}+\mu u^{2}\right)r^{2}}drdu.$$

To continue deriving (36), refer as follows:

(37) $$\begin{array}{c} \int_{-\infty }^{\infty }x^{2}e^{-ax^{2}}dx=-\frac{d}{da}\int_{-\infty }^{\infty }e^{-ax^{2}}dx\\ =-\frac{d}{da}\left[a^{-\frac{1}{2}}\underset{=\sqrt{\pi }}{\underset{︸}{\int_{-\infty }^{\infty }e^{-x^{2}}dx}}\right]=\frac{\sqrt{\pi }}{2a^{\frac{3}{2}}}, \end{array}$$

(38) $$\int_{0}^{\infty }x^{2}e^{-ax^{2}}dx=\frac{\sqrt{\pi }}{4a^{\frac{3}{2}}}.$$

Therefore, (36) will be:

(39) $$\begin{array}{c} \frac{\sqrt{\mu }\sigma_{x}^{3}}{2(\sigma_{x}^{2}-\sigma_{y}^{2})}\int_{1}^{\infty }\frac{1}{{\left(1+2\sigma_{x}^{2}\mu u^{2}\right)}^{\frac{3}{2}}}du. \end{array}$$

To continue deriving (39), substitute proceeds as follows:

(40) $$u=\sqrt{\frac{1}{2\sigma_{x}^{2}\mu }}tan\theta ,\;\frac{du}{d\theta }=\sqrt{\frac{1}{2\sigma_{x}^{2}\mu }}sec^{2}\theta ,$$

(41) $$\begin{array}{c} \frac{\sigma_{x}^{2}}{2(\sigma_{x}^{2}-\sigma_{y}^{2})}\int_{tan^{-1}\left(\sqrt{2\sigma_{x}^{2}\mu }\right)}^{\frac{\pi }{2}}cos\theta d\theta \\ =\frac{\sigma_{x}^{2}}{2(\sigma_{x}^{2}-\sigma_{y}^{2})}\left[1-sin\left(tan^{-1}\left(\sqrt{2\sigma_{x}^{2}\mu }\right)\right)\right], \end{array}$$

(42) $$sin\theta =\sqrt{\frac{tan^{2}\theta }{1+tan^{2}\theta }},$$

(43) $$sin\left(tan^{-1}\left(\sqrt{2\sigma_{x}^{2}\mu }\right)\right)=\sqrt{\frac{2\sigma_{x}^{2}\mu }{1+2\sigma_{x}^{2}\mu }}.$$

Therefore:

(44) $$\frac{\sigma_{x}^{2}}{2(\sigma_{x}^{2}-\sigma_{y}^{2})}\left(1-\sqrt{\frac{2\sigma_{x}^{2}\mu }{1+2\sigma_{x}^{2}\mu }}\right).$$

Based on (44), finally, Equation (33) can be expressed as follows:

(45) $$\begin{array}{c} \int_{0}^{\infty }p_{df}\left(r\right)p_{bpsk}\left(e\right)dh=\frac{\sigma_{x}^{2}}{2(\sigma_{x}^{2}-\sigma_{y}^{2})}\left(1-\sqrt{\frac{2\sigma_{x}^{2}\mu }{1+2\sigma_{x}^{2}\mu }}\right)\\ -\frac{\sigma_{y}^{2}}{2(\sigma_{x}^{2}-\sigma_{y}^{2})}\left(1-\sqrt{\frac{2\sigma_{y}^{2}\mu }{1+2\sigma_{y}^{2}\mu }}\right). \end{array}$$

By adopting (45) to (32), $\epsilon_{2}$ can be calculated in the closed-form.

Therefore, the final BLER α considering both the first transmission $\epsilon_{1}$ and retransmission $\epsilon_{2}$, is:

(46) $$\alpha =\epsilon_{1}\times \epsilon_{2}.$$

#### 3.1.2. Practical Scenario

The practical scenario deals with I-CSI and I-SIC. To consider I-CSI in the first transmission:

(47) $$\begin{array}{cc} \; & y=h\sqrt{E_{b}}+N_{0}\\ \; & =\hat{h}\sqrt{E_{b}}+e\sqrt{Eb}+N_{0}\\ \; & =\hat{h}\sqrt{E_{b}}+\hat{n}, \end{array}$$

where *e* is the channel estimation(CE) error, $\hat{n}$ is a noise including CE error.

(48) $$\hat{\sigma }=\sqrt{\sigma^{2}-\sigma_{e}^{2}},\;\hat{\mu }=\frac{E_{b}}{2(E_{b}\sigma_{e}^{2}+\sigma_{n}^{2})},$$

where σ is the standard deviation of fading channel, $\sigma_{e}$ is the standard deviation of CE error, and $\sigma_{n}$ is $\frac{N_{0}}{2}$.

Based on (16) and (48), σ and μ are substituted. Then, the BER p(e) is as follows:

(49) $$\begin{array}{c} p\left(e\right)=\frac{1}{4}(2-\sqrt{\frac{2{\hat{\sigma }}^{2}{\left(p_{n}+p_{f}\right)}^{2}\hat{\mu }}{1+2{\hat{\sigma }}^{2}{\left(p_{n}+p_{f}\right)}^{2}\hat{\mu }}}\\ -\sqrt{\frac{2{\hat{\sigma }}^{2}{\left(p_{f}-p_{n}\right)}^{2}\hat{\mu }}{1+2{\hat{\sigma }}^{2}{\left(p_{f}-p_{n}\right)}^{2}\hat{\mu }}}), \end{array}$$

(50) $$\begin{array}{c} p\left(e\right)=\frac{1}{4}(2-\sqrt{\frac{\left(\sigma^{2}-\sigma_{e}^{2}\right){\left(p_{n}+p_{f}\right)}^{2}}{\sigma_{e}^{2}+\sigma_{n}^{2}+\left(\sigma^{2}-\sigma_{e}^{2}\right){\left(p_{n}+p_{f}\right)}^{2}}}\\ -\sqrt{\frac{\left(\sigma^{2}-\sigma_{e}^{2}\right){\left(p_{f}-p_{n}\right)}^{2}}{\sigma_{e}^{2}+\sigma_{n}^{2}+\left(\sigma^{2}-\sigma_{e}^{2}\right){\left(p_{f}-p_{n}\right)}^{2}}}). \end{array}$$

Then, we can get the BLER of the first transmission $\epsilon_{1}$:

(51) $$\epsilon_{1}=1-{\left(1-p\left(e\right)\right)}^{n}.$$

Equation (51) can also be used for SIC error rate by just substitute σ to the standard deviation of fading channel between BS and CCU, $\sigma_{z}$. If SIC succeeds, the CCU can retransmit the signal to the CEU. However, if the CCU fails SIC, not only the CCU cannot decode the signal for itself but also cannot retransmit the signal for the CEU. Therefore, the error rate of the retransmission should be considered whether SIC successes or not. Based on (51), the SIC error rate can be calculated as follows:

(52) $$\epsilon_{SIC}=1-{\left(1-p_{SIC}\left(e\right)\right)}^{n},$$

where $\epsilon_{SIC}$ is the SIC error rate of CCU and $p_{SIC}\left(e\right)$ the BER of CCU on a bit for the CEU.

(53) $$\begin{array}{c} p_{SIC}\left(e\right)=\frac{1}{4}(2-\sqrt{\frac{\left(\sigma_{z}^{2}-\sigma_{e}^{2}\right){\left(p_{n}+p_{f}\right)}^{2}}{\sigma_{e}^{2}+\sigma_{n}^{2}+\left(\sigma_{z}^{2}-\sigma_{e}^{2}\right){\left(p_{n}+p_{f}\right)}^{2}}}\\ -\sqrt{\frac{\left(\sigma_{z}^{2}-\sigma_{e}^{2}\right){\left(p_{f}-p_{n}\right)}^{2}}{\sigma_{e}^{2}+\sigma_{n}^{2}+\left(\sigma_{z}^{2}-\sigma_{e}^{2}\right){\left(p_{f}-p_{n}\right)}^{2}}}), \end{array}$$

where *z* is the channel state between BS and CCU.

If the SIC is failed, only the BS will retransmit. Therefore, BER for retransmission only from BS, $p_{re}\left(e\right)$, is as follows:

(54) $$\begin{array}{c} p_{re}\left(e\right)=\frac{1}{2}\left(1-\sqrt{\frac{2{\hat{\sigma }}_{x}^{2}\hat{\mu }}{1+2{\hat{\sigma }}_{x}^{2}\hat{\mu }}}\right)\\ =\frac{1}{2}\left(1-\sqrt{\frac{\sigma_{x}^{2}-\sigma_{e}^{2}}{\sigma_{n}^{2}+\sigma_{x}^{2}}}\right). \end{array}$$

Then, the BLER of retransmission $\epsilon_{2f}$ in the case of failed SIC is as follows:

(55) $$\epsilon_{2f}=1-{\left(1-p_{re}\left(e\right)\right)}^{n}.$$

If the SIC success, both the BS and the CCU will retransmit the signal to CEU. Based on (45) and (48), the BER for retransmission from BS and CCU simultaneously, $p_{re}\left(e\right)$, is as follows:

(56) $$\begin{array}{c} p_{re}\left(e\right)=\frac{{\hat{\sigma }}_{x}^{2}}{2({\hat{\sigma }}_{x}^{2}-{\hat{\sigma }}_{y}^{2})}\left(1-\sqrt{\frac{2{\hat{\sigma }}_{x}^{2}\hat{\mu }}{1+2{\hat{\sigma }}_{x}^{2}\hat{\mu }}}\right)\\ -\frac{{\hat{\sigma }}_{y}^{2}}{2({\hat{\sigma }}_{x}^{2}-{\hat{\sigma }}_{y}^{2})}\left(1-\sqrt{\frac{2{\hat{\sigma }}_{y}^{2}\hat{\mu }}{1+2{\hat{\sigma }}_{y}^{2}\hat{\mu }}}\right), \end{array}$$

(57) $$\begin{array}{c} p_{re}\left(e\right)=\frac{\sigma_{x}^{2}-\sigma_{e}^{2}}{2(\sigma_{x}^{2}-\sigma_{y}^{2})}\left(1-\sqrt{\frac{\sigma_{x}^{2}-\sigma_{e}^{2}}{\sigma_{x}^{2}+\sigma_{n}^{2}}}\right)\\ -\frac{\sigma_{y}^{2}-\sigma_{e}^{2}}{2(\sigma_{x}^{2}-\sigma_{y}^{2})}\left(1-\sqrt{\frac{\sigma_{y}^{2}-\sigma_{e}^{2}}{\sigma_{y}^{2}+\sigma_{n}^{2}}}\right). \end{array}$$

Then, the BLER of retransmission $\epsilon_{2s}$ in the case of succeeded SIC is as follows:

(58) $$\epsilon_{2s}=1-{\left(1-p_{re}\left(e\right)\right)}^{n}.$$

Therefore, the final BLER α is:

(59) $$\alpha =\epsilon_{1}\times \left(\epsilon_{SIC}\times \epsilon_{2f}+\left(1-\epsilon_{SIC}\right)\times \epsilon_{2s}\right).$$

### 3.2. PLR of Cooperative Retransmission Scheme

**Figure 3:**
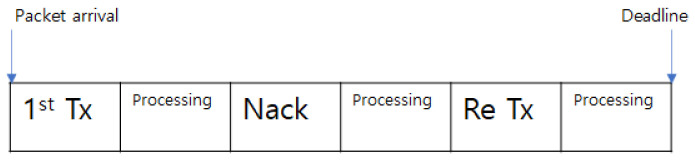
Retransmission process.

In this subsection, packet loss rate (PLR), which includes both cases that the packet is dropped and lost until the latency constraint, is expressed based on the cumulative binomial distribution. Figure 3 describes how the URLLC packet will be processed in this system. When a URLLC packet for a CEU is generated, the packet should be transmitted in the first time slot to satisfy the latency constraint. Subsequently, after one time slot for processing in the CEU, the CEU transmits ack/nack to BS. Next, after one time slot for processing, BS, and CCU will simultaneously retransmit parts of the packet corrupted in the first transmission. At this time, when the retransmission is underway, the BS and the CCU retransmit the signal on the same resource blocks. Subsequently, the CEU processes the packet retransmitted in the last timeslot before the deadline. Therefore, PLR includes the first transmission and retransmission until the deadline with limited resource blocks. If the number of resource blocks is not enough to cover whole generated packets including first transmissions and retransmissions, packets will be dropped, and packet losses will be counted. And if the retransmission packet is corrupted without a shortage of resource blocks, packet loss will also be counted. In this process, if a packet misses the timing for the first transmission or retransmission due to a resource block shortage, the packet will be automatically dropped. This is because the packet cannot satisfy the latency constraint if the packet misses the transmission within the given timeslot.

The conditions of packet losses are below:

- The number of resource blocks is not enough for allocating the generated whole packets.
- Some parts of the packets are corrupted owing to interference, noise, and bad channel state in both first transmission and retransmission.

Therefore, first, the probability that the number of resource blocks is enough to cover packets of CEUs should be calculated. Then, the reliability considering both the first transmission and retransmission needs to be calculated. Based on the calculation, PLR until the latency constraint can be derived. Allocating packets on resource blocks can cause a lack of resource blocks for other packets. Therefore, the resource blocks allocated for packets should be calculated to obtain the PLR. The resource blocks in the current time slot comprise retransmissions of packets that were generated in the previous timeslot which means three timeslots earlier than the current timeslot, and first transmissions of packets currently appear. $\alpha_{1}$, the probability of how many *j* retransmissions occur by the previous packets is given below:

(60) α1=∑i=0NNi(λ)i(1−λ)N−i︸A×∑j=0sisij(ϵ1)j(1−ϵ1)si−j,ifsi≤r∑j=0rrj(ϵ1)j(1−ϵ1)r−j,ifsi>r︸B.

*N* denotes the number of CEUs, λ is the packet arrival rate, $\epsilon_{1}$ is the BLER of the first transmission, *s* is the number of required resource blocks for the packet, and *r* is the number of total resource blocks allocated for CEUs. By $\alpha_{1}$ in (60), the probability of how many retransmissions are allocated in the current timeslot can be calculated. Term A is the probability of *i* packets arrival in the previous timeslot that is three timeslots earlier. Term B is the probability how many block error came out in the previous timeslot.

Subsequently, the probability of how many first transmissions are generated fewer than resource blocks subtracted by the number of *j* retransmissions from the previous timeslot should be calculated. *z* in (61) denotes the number of possible users who can transmit with the remaining resource blocks after allocating retransmissions on resource blocks. It can be calculated by rounding down $\frac{r-j}{s}$,

(61) $$z=\lfloor \frac{r-j}{s}\rfloor .$$

In the case of the packets generated in the current timeslot, considering the transmission success probability $\alpha_{2}$ is as follows:

(62) α2=∑k=0N−1N−1k(λ)k(1−λ)N−1−k︸C×zk+1,ifz<k+11,ifz≥k+1︸D×∑l=0ssl(ϵ1)l(1−ϵ1)s−l︸E×(1−ϵ2)l︸F.

$\epsilon_{2}$ is the BLER for retransmission.

Term C is the probability that the other packets arrival when one packet arrivals in the current timeslot. Term D is the probability that the packet is selected among the whole generated packets. Term E is the probability that *l* blocks of the packet are corrupted. Term F is the probability that retransmissions of *l* blocks are successful. Then, according to choosing $\epsilon_{2}$ as the CR scheme or the conventional scheme, the PLR performance will be different. Finally, the PLR ε is as follows,

(63) $$\varepsilon =1-\alpha_{1}\times \alpha_{2}$$

## 4. Performance Evaluations

In this section, the performance of the CR scheme is evaluated in terms of reliability and latency constraint.

**Figure 4:**
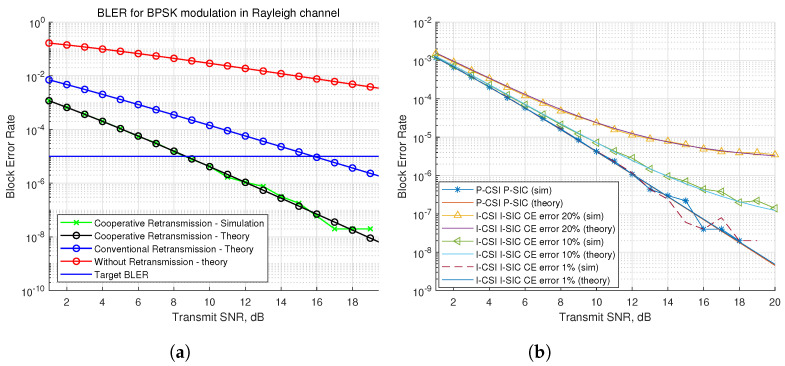
Block error rate comparison. (**a**) Ideal scenario. (**b**) Practical scenario.

Parameters for BLER evaluations are in Table 1. Figure 4a shows the BLER performance for a CEU in the ideal scenario, which assumes P-SIC and P-CSI. When the transmit SNR is 9 dB, our proposed retransmission scheme meets the target BLER. The others show inferior performance compared to our proposed scheme. The conventional retransmission scheme, which is from only the BS, arrives at the target BLER when the transmit SNR is 16 dB. In the case of no retransmission, the target BLER cannot be reached. This means that using the only NOMA without retransmission makes it difficult to ensure the reliability of URLLC.

Figure 4b shows the BLER performance in the practical scenario, which assumes I-SIC and I-CSI. Compared to the ideal scenario, we can see CE error affects the BLER results. When the CE error is 20%, the BLER becomes the highest compared to the other results. When the CE error is 1%, it performs similar results to the ideal scenario. As a matter of course, reducing CE error is an important part to improve the reliability of users in this system. In addition, in high SNRs, even though noise levels become smaller than in low SNRs, the improvements of BLER performance of 10% and 20% CE error becomes not sharp, because the residual CE error weakens the BLER performance.

**Figure 5:**
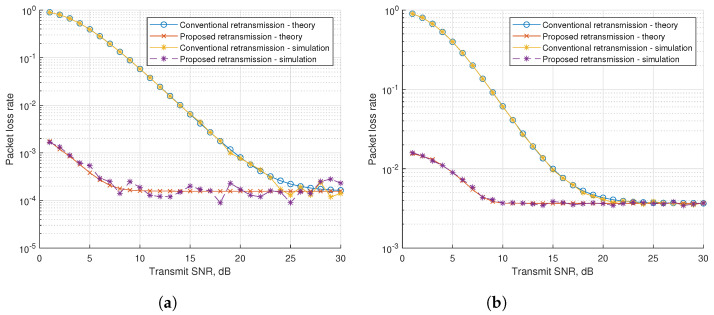
Packet loss rate with fixed packet arrival rates. (**a**) Packet arrival rate 0.1. (**b**) Packet arrival rate 0.2.

For PLR simulation, the simulation parameters are shown in Table 2. Figure 5a,b show the results according to varying transmit SNR when the packet arrival rates are 0.1 and 0.2, respectively. The CR scheme shows better performance than the conventional retransmission in simulations, because the CR scheme has higher reliability in the case of retransmission. On increasing transmit SNR, the gap between the conventional retransmission and proposed schemes becomes narrow. As the error rate will be lower owing to the transmit SNR getting improved along the X-axis, the number of retransmissions reduces and the probability of the transmission success increases.

**Figure 6:**
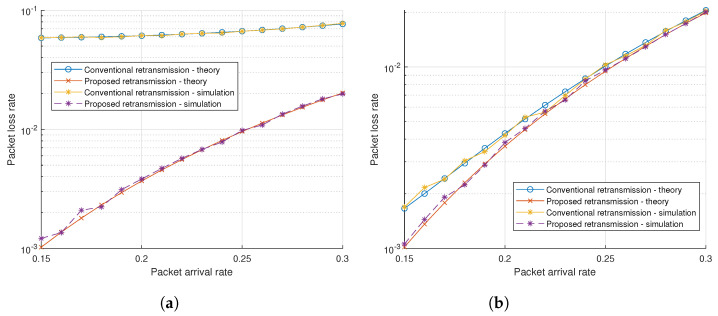
Packet loss rate with fixed transmit SNR. (**a**) Transmit SNR 10 dB. (**b**) Transmit SNR 20 dB.

Figure 6a,b show the results of varying packet arrival rates when the transmit SNRs are 10 and 20 dB, respectively. In these cases, the proposed scheme shows better performance than the conventional schemes. However, the greater the increase in the packet arrival rate, the narrower is the gap between the conventional retransmission scheme and the proposed scheme because the congestion will arise more frequently due to more packets generated.

## 5. Conclusions

In this study, a cooperative retransmission scheme in multicarrier downlink NOMA systems was investigated, and analytical models and simulation results for BLER and PLR were realized. The proposed scheme was evaluated through comparisons with the conventional retransmission scheme in terms of the reliability and latency constraints of URLLC, and it demonstrated the proposed scheme satisfied the reliability requirement of URLLC at 9 dB in BLER performance, however, the others cannot satisfy the reliability requirement. And the evaluation of BLER for the practical scenario, which considers imperfect CSI and SIC, was performed with the different CE errors. The effect of CE errors determined that the differences with different CE errors are clearly shown in high SNRs. And PLR performance was evaluated comparing CR scheme to conventional retransmission scheme. As the CR scheme has a lower error rate than of conventional retransmission scheme, the CR scheme arrived at the lowest PLR faster than the conventional scheme along transmit SNRs. However, increasing SNR cannot show the improvement of CR scheme in high SNR, because the limitation of resource blocks causes congestion causing packet drop. Future works should focus on adopting the proposed retransmission scheme with a finite blocklength regime [16] in uplink NOMA systems.

## Figures and Tables

**Table 1:** BLER Simulation parameters.

The antenna on the BS and users	Single
Power allocation ratio for the CCU	0.2
Power allocation ratio for the CEU	0.8
Channel model	Quasi-static rayleigh fading
Path loss exponent	4
Target error rate	$10^{-5}$
Normalized distance between BS and CEU	0.9
Normalized distance between CCU and CEU	0.7
Normalized distance between BS and CCU	0.2

**Table 2:** PLR simulation parameters.

Channel estimation error	0.1
Normalized distance between the CCUs and BS	0.1–0.3
Normalized distance between the CEUs and BS	0.8–1.0
Power allocation ratio for the CCUs	0.2
Power allocation ratio for the CEUs	0.8
Bandwidth	10 MHz
Sub-band bandwidth	15 kHz
The number of subcarriers	666
Transmission time interval(TTI)	0.144 ms
TTI duration	2 symbols
Channel model	Quasi-static Rayleigh fading
Path loss exponent	4
Target error rate	$10^{-5}$
Latency constraint	0.864 ms (6 timeslots)
URLLC packet size	32 bytes
Simulation time	1.44 × $10^{5}$ ms
The number of BS	1
The number of user pairs	10

## Data Availability

Data sharing is being prepared.
